# Birth-death prior on phylogeny and speed dating

**DOI:** 10.1186/1471-2148-8-77

**Published:** 2008-03-04

**Authors:** Örjan Åkerborg, Bengt Sennblad, Jens Lagergren

**Affiliations:** 1 Stockholm Bioinformatics Centre (SBC), Albanova, Stockholm University, SE-10691 Stockholm, Sweden; 2 School for Computer Science and Communication (CSC), Royal Institute of Technology (KTH), SE-10044 Stockholm, Sweden

## Abstract

**Background:**

In recent years there has been a trend of leaving the strict molecular clock in order to infer dating of speciations and other evolutionary events. Explicit modeling of substitution rates and divergence times makes formulation of informative prior distributions for branch lengths possible. Models with birth-death priors on tree branching and auto-correlated or *iid *substitution rates among lineages have been proposed, enabling simultaneous inference of substitution rates and divergence times. This problem has, however, mainly been analysed in the Markov chain Monte Carlo (MCMC) framework, an approach requiring computation times of hours or days when applied to large phylogenies.

**Results:**

We demonstrate that a hill-climbing maximum *a posteriori *(MAP) adaptation of the MCMC scheme results in considerable gain in computational efficiency. We demonstrate also that a novel dynamic programming (DP) algorithm for branch length factorization, useful both in the hill-climbing and in the MCMC setting, further reduces computation time. For the problem of inferring rates and times parameters on a fixed tree, we perform simulations, comparisons between hill-climbing and MCMC on a plant *rbcL *gene dataset, and dating analysis on an animal mtDNA dataset, showing that our methodology enables efficient, highly accurate analysis of very large trees. Datasets requiring a computation time of several days with MCMC can with our MAP algorithm be accurately analysed in less than a minute. From the results of our example analyses, we conclude that our methodology generally avoids getting trapped early in local optima. For the cases where this nevertheless can be a problem, for instance when we in addition to the parameters also infer the tree topology, we show that the problem can be evaded by using a simulated-annealing like (SAL) method in which we favour tree swaps early in the inference while biasing our focus towards rate and time parameter changes later on.

**Conclusion:**

Our contribution leaves the field open for fast and accurate dating analysis of nucleotide sequence data. Modeling branch substitutions rates and divergence times separately allows us to include birth-death priors on the times without the assumption of a molecular clock. The methodology is easily adapted to take data from fossil records into account and it can be used together with a broad range of rate and substitution models.

## Background

Since Felsenstein brought the maximum likelihood (ML) framework to phylogenetic inference [1] the number of supporters of likelihood based estimation has steadily increased, and it is now widely considered the most accurate approach. Although ML phylogenetic inference is generally quicker than stochastic optimization inference (MCMC), the time complexity also of ML-algorithms has been prohibitive for analysis of large phylogenies. Recently, however, the field has seen considerable advances in speed of the best methods, among which PHYML [2] and RAxML [3] are notable, seemingly without sacrificing much accuracy. With these methods phylogenies with tens or even hundreds of taxa [2] are readily examined. After its introduction a decade ago [4-6], the use of Bayesian methods in phylogenetic inference has been a field of active research in which not only the phylogeny itself has been sought, but also additional issues have been addressed, such as substitution rate hypotheses, accuracy of ancestral state inference, and the rooting problem, see [7] for a review. In particular, a number of alternatives to the much debated molecular clock hypothesis [8] have been suggested. Among these are models with molecular clocks operating locally [9,10] and also a range of models with rate variation over lineages; these include auto-correlated models, i.e., the rate distribution for a particular branch depends on the rate value of the parent branch [11-15], and uncorrelated models where rates are drawn independently from a common underlying distribution [16] (Sennblad et al.: Parental guidance vs. mutual independence – evaluation of bayesian models of substitution rate evolution, submitted).

A fundamental question for the accuracy of Bayesian phylogenetic inference is the selection of a prior distribution on trees and branch lengths [17], and several types of such have been suggested. An intuitively appealing choice is to assume that the tree branching follows a birth-death process which is indeed what is exploited by Yang and Rannala [4,18]. In [18], the authors assumed a molecular clock and used integration over branching times to calculate the posterior distribution of tree topologies, a procedure they reported infeasible for trees with more than five leaves. In order to overcome this, they applied an MCMC methodology to the problem [4], but the time complexity of the algorithm still severely limited the number of taxa that could be analyzed.

One might think that a model containing a birth-death prior on the tree branching would necessarily be consistent with a molecular clock, since the birth-death process generates ultrametric trees. The molecular clock can be avoided, however, by modeling the substitution rates and branching times separately. Biologically, the ability to separately infer rates and times is of importance, since the former can pinpoint periods of molecular function changes and the latter enables dating of speciations and other evolutionary events. Moreover, as was recognized by Arvestad *et al. *[19,20], explicit time modeling facilitates integration of separate time dependent evolutionary models into a unified framework.

In a paper on MCMC-estimation of phylogenies and divergence times [16], the authors performed simulations comparing uncorrelated and clock-like models, and concluded that clock-like models only perform well on clock-like data, while uncorrelated models always perform acceptably. Furthermore, they investigated parameters inferred from two viral and one marsupial dataset with the result that no significant auto-correlation was detected. One conclusion in the paper was, therefore, that auto-correlated models are not generally suitable. This contrasts with the results presented in a recent paper by Lepage *et al. *[21]. They evaluate two auto-correlated models and concludes that they clearly outperform the uncorrelated models tested.

In the work presented here, we make use of a Bayesian framework with informative priors on branch lengths. We have investigated solutions to the *parameter inference problem*, i.e., inference of rates and times, as well as the *phylogeny inference problem*, where we also infer the tree topology. These problems are analysed with a hill-climbing maximum *a posteriori *(MAP) as well as an MCMC methodology. We further introduce a dynamic programming (DP) algorithm for optimal factorization of branch lengths into rates and times, thereby considerably reducing the computation time needed for hill-climbing as well as MCMC-algorithms. The nucleotide substitution is modeled by a continuous-time Markov process and we use a birth-death process to obtain an *a priori *distribution of phylogenies' branching times. The substitution rates are drawn *iid *from a Γ-distribution [22]. We perform simulations to show that with our method fast simultaneous inference of substitution rates and branching times for a given tree topology is not only feasible on large trees but also largely unaffected by local-optima problems. By comparing our results with MCMC-algorithms based on, in one case, the same model (Sennblad et al.: Parental guidance vs. mutual independence – evaluation of bayesian models of substitution rate evolution, submitted), and in another case, a similar model [23], we show that despite its uncomplicated nature, the presented algorithm delivers parameter estimations with high accuracy. Finally, we show that also the phylogeny inference problem is manageable in acceptable time. By using a simulated annealing-inspired methodology, the *simulated annealing like (SAL)-method*, where tree topologies are swapped often in the beginning, but henceforth more rarely, we can avoid getting stuck on a particular local optimum tree.

## Methods

### General notation

Let *s *be the number of aligned sequences and *n *their common length with columns of indels omitted. The data will be represented by an *s *× *n *matrix *D *= {*d*_*ij*_}. The topology of the phylogenetic tree relating the sequences is denoted by *T*. Assuming the nucleotide substitution rate to be constant on any particular edge in the tree, we will denote the length, the rate, and the time of the edge connecting node *v *with its parent by *l*_*v*_, *r*_*v*_, and *t*_*v*_, respectively, where *l*_*v *_≡ *r*_*v*_*t*_*v*_. We will refer to the complete vectors of lengths, rates, and times, for all edges as **l**, **r**, and **t**, respectively.

The discussion in this paper centers around *P *[**r**, **t**|*D*, *T*], the probability of the rates and the times given the sequences and the tree topology, and *P *[**r**, **t**, *T*|*D*], the probability of the rates, the times, and the tree topology given the sequences. *P *[**r**, **t**|*D*, *T*] is defined by:

(1) $$P[r,t|D,T]=\frac{\int P[D|r,t,T]p[r]p[t|T]drdt}{P[D]}.$$

We will refer to the factor *P *[*D*|**r**, **t**, *T*] as the *data probability *and to factors *p *[**r**] and *p *[**t**|*T*] as *rates prior *and *times prior*, respectively. The probability *P *[**r**, **t**|*D*, *T*] will be referred to as the *posterior*. The value of the data probability depends solely on the tree's branch lengths whereas the product of the rates and the times prior probabilities, which can be viewed as informative length priors, depends on how these lengths are factorized into rates and times.

### Three solutions to the parameter inference problem

The dimensionality of the integral in (1) is (2*s *- 2) + (*s *- 2) for a tree inferred from *s *sequences, making its numerical evaluation feasible only for relatively small *s *[18]. Performing the integration by means of MCMC is the natural remedy, but this is a notoriously computationally intensive methodology. In the following, we will therefore study several aspects of the alternative problem of finding the corresponding MAP solution:

(2) $$\underset{r,t}{\arg\max}P[D|r,t,T]p[r]p[t|T],$$

namely, the most likely complete set of rates and times given the data and the model. We have implemented three algorithms for finding MAP solutions. As a first alternative, the ***r ***× ***t***-*method*, we explore the entire **r **× **t**-space and seek the optimum in that space. As a second alternative, the ***l***-*method*, we search the **l**-space and when a supposed **l**-optimum is found, we factorize the lengths into rates and times. Neglecting issues regarding non-global optima, we expect the **l**-method to find a solution that is optimal only with regard to the data probability, while the **r **× **t**-method should eventually find the optimal solution including the priors on rates and times (i.e. with the same optimal data probability but a better prior probability). Unfortunately, the **r **× **t**-method is impractically slow. To overcome the weaknesses of these two methods, we have developed a DP algorithm, to be described below, which finds the best possible factorization of any given length vector **l **under the rates and times priors.

By applying our DP algorithm, we can optimize the factorization of the lengths resulting from the **l**-space search. Even so, we may very well end up in a situation where we have found an optimal **l **but where there is another **l **with nearly as good data probability but prior value so superior that the posterior is better. That solution cannot be found with the **l**-method. By instead combining the DP and the **r **× **t**-algorithms, it is possible to achieve a better result in a reasonable time. We search the **r **× **t**-space as before, but at each iteration, the search is interrupted with probability *p *(we have found *p *= 0.001 to be suitable). If so, we factorize the current **l **optimally, and make a desirable jump in the search space that would otherwise have been impossible. This method will be referred to as the *combined *method.

### A DP algorithm for branch length factorization

**Figure 1:**
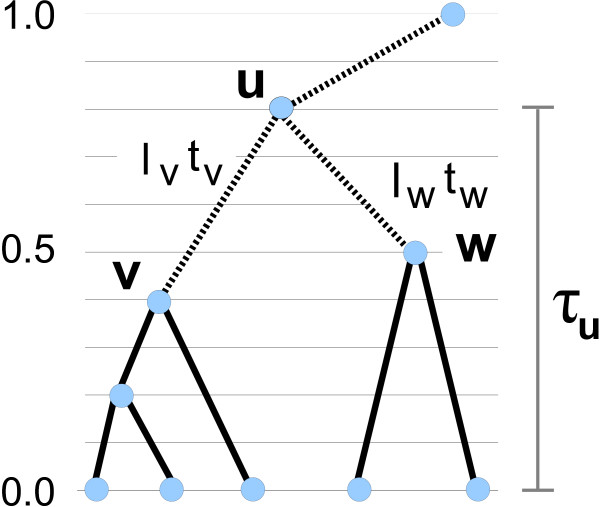
**A dynamic programming algorithm for branch length factorization**. Values *f*_*u*_(*τ*_*u*_) are calculated for all possible choices of *τ*_*u*_. *τ*_*u *_is *u*'s divergence time counted from the leaves and it takes values corresponding to the equidistant grid. *f*_*u*_(*τ*_*u*_) is a product of rate priors for the edges leading to *u*, *u*'s contribution to the time prior, and the corresponding probabilities for *u*'s children *v *and *w*: *f*_*v*_(*τ*_*u*_) and *f*_*w*_(*τ*_*u*_) respectively.

Given lengths for each edge in the tree, the objective of the DP algorithm is to factorize the lengths into optimal rate and time parameter estimates for the edges. To facilitate this, a discretization of the time interval from the leaves to the root is made. We scale this interval in order to give the leaf times and the root times values zero and one, respectively. All *s *- 2 non-root inner nodes are assigned intermediate values corresponding to the equidistant grid that is the result of the discretization (see Figure 1). The number of grid intervals is *N *(we have found *N *= 100 to be suitable). For a given node *u *with children *v *and *w*, *f*_*u*_(*t*_*u*_), denoting *p *[*r*]*p *[*τ*_*u*_|*T*_*u*_], is calculated for each possible discretized divergence time *τ*_*u *_of *u*, using all possible discretized divergence times for *v *and *w*. For the node *u*, *f*_*u*_(*τ*_*u*_) is given by the maximum value over *τ*_*v*_, *τ*_*w *_of:

(3) $$\underset{\text{rate priors}}{\underset{︸}{p[l_{v}/t_{v}]p[l_{w}/t_{w}]}}\underset{\begin{array}{l} \text{time prior}\\ \text{contribution} \end{array}}{\underset{︸}{p[\tau_{u}|T_{u},\tau_{v},\tau_{w}]}}f(\tau_{v})f(\tau_{w})$$

where *t*_*v *_≡ *τ*_*u *_- *τ*_*v *_and *t*_*w *_≡ *τ*_*u *_- *τ*_*w*_. The values of the rate priors are dependent on the gamma distribution and the quotient between the lengths and the times for the edges leading to *u*. We use the prior densities for branching times given a tree, derived in [24]. This yields values dependent on the birth-death process, *τ*_*u*_, and the number of leaves of the subtree *T*_*u *_in which *u *is the root. We work from the leaves up and, when we reach the root, the optimal *f*_*root*_(1.0) ≡ *p *[**r**]*p *[**t**|*T*] and the rate-time factorization of the lengths that gave rise to the optimal value are retrieved.

### Rate model and hyperparameter values

In this study, we use a rate model described in (Sennblad et al.: Parental guidance vs. mutual independence – evaluation of bayesian models of substitution rate evolution, submitted). Briefly, this model averages the rate for each branch as independent and identically distributed (iid) stochastic variables drawn from an underlying Γ-distribution. Apart from the edge rates themselves, the model has two *hyperparameters*, namely the mean *m *and the variance *v *of the Γ-distribution, which are both assigned uniform priors in the interval [0, 1000]. Variants of the iid model with respect to the underlying distribution (e.g., 3, Log Normal, Inverse Gaussian and Exponential distributions) have been described [16]. A comparative study in (Sennblad et al.: Parental guidance vs. mutual independence – evaluation of bayesian models of substitution rate evolution, submitted) found no difference in performance between these variants, while results in [21] suggested that the Γ-distribution might be more flexible in describing rate variation. An alternative approach suggested by Sanderson [12,13] and Thorne *et al. *[14], is to model rates as evolving over the tree, such that there is an auto-correlation between rates at adjacent nodes. In (Sennblad et al.: Parental guidance vs. mutual independence – evaluation of bayesian models of substitution rate evolution, submitted), the differences in performance of the iid model and the evolving model were found to be negligible. In [16], the authors argue that over short time scales inherited factors become small relative to stochastic factors, and that over very long time scales the variation causes the auto-correlation from lineage to lineage to break down.

In the recent paper by Lepage *et al. *[21], the authors perform benchmarking of relaxed clock models. They test various choices of divergence time priors (birth-death, Dirichlet, uniform) and variants of both auto-correlated and uncorrelated rate models. This test favors the auto-correlated rate model alternative while the optimal choice of time prior is found to be more data dependent.

In this study, we have chosen the iid-Γ model as an example distribution; it is, however, straight-forward to adapt our methodology to accommodate any of the models described above, with a possible exception for the Dirichlet time prior which we have not tested.

The birth-death process generating tree branching times have hyperparameters for birth rate, *λ*, and death rate, *μ*, respectively. That is, the set of hyperparameters used in the inference are *λ*, *μ*, *m*, and *v*. For the simulation analyses, we generated sequences using *λ *= 1.0, *μ *= 1.0, *m *= 0.5, *v *= 0.01. We then used these same values during inference. We used the simple Jukes-Cantor [25] substitution model. When analyzing biological data, we estimated values for *λ*, *μ*, *m*, *v *during inference and the substitution parameters were estimated with a maximum likelihood analysis using PAUP [26] and the GTR+Γ model [27].

### Proposal distributions

The proposal scheme used was developed for the MCMC-algorithm described in (Sennblad et al.: Parental guidance vs. mutual independence – evaluation of bayesian models of substitution rate evolution, submitted). Each parameter (i.e. rates, times, and model hyperparameters) has equal probability of being updated, and the new value *s*' of the updated parameter *s *is *LogNormal*(*s*, *σ*). The factor *σ *is a prespecified constant related to the parameter type which was calibrated to optimize mixing and convergence of the MCMC chain.

We present here results obtained with MCMC, as well as a hill-climbing MAP adaptation of the MCMC-algorithm. In the MAP case we only accept proposals increasing the posterior, while in the MCMC case the standard Metropolis-Hastings proposal-acceptance scheme is used.

We further tested a deterministic scheme where a search was performed in all directions, i.e., we perturb each parameter, store all results and then choose the perturbation giving the optimal log-likelihood. In this case, the results were qualitatively the same as with the randomized algorithm described above, although computation times were longer.

### The phylogeny inference problem and the SAL-method

When we extend our ambition to also do phylogeny inference, the most severe problem with the hill-climbing approach is that the algorithm can get stuck on a particular non-global optimum tree. This problem can, at least to some extent, be resolved by the SAL-method, i.e., a methodology where tree topologies are swapped often in the beginning, but henceforth more rarely. For simplicity, we use a linear scheme where the probability of a tree swap is originally *k *times higher than that of a parameter change (we have found *k *= 1000 to be suitable). For each tree swap attempt, we decrease *k *with one unit until *k *equals unity. The types of tree rearrangements that we use are re-rooting, nearest neighbor interchange (NNI) and subtree pruning and regrafting (SPR), see [7].

The applicability of the DP-algorithm as well as the SAL-method are independent of the way in which we exploit the search space, i.e., they are useful in both the MAP and the MCMC setting.

## Results

### Parameter inference simulations

**Figure 2:**
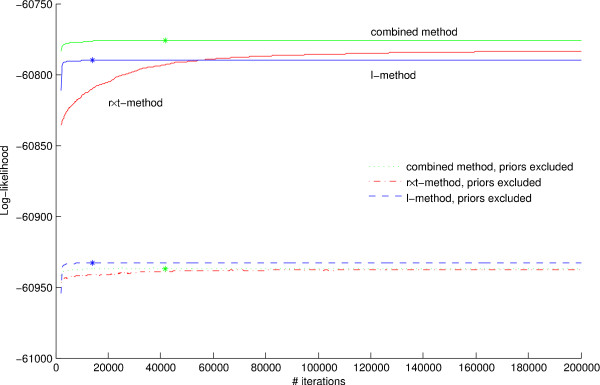
**Three MAP inference methods – a comparison**. The plot illustrates the fact that optimization over the full **r **× **t **parameter space (red curves) is much slower than both the equivalent optimization over **l **with subsequent DP-partition of **l **into **r **and **t **(blue curves), and the method combining **r **× **t **search with occasional DP-optimization interruptions (green curves). The solid curves show log-likelihoods including the priors *p *[*r*]*p *[*t*|*T*], whereas the dashed curves show the same results, but excluding that factor. Approximate point of convergence, i.e., the first point when 1000 subsequent steps has not resulted in any improvement, is marked with an asterisk.

We first evaluated the three inference methods described above on fixed trees with respect to their capacity to infer parameters. We generated a tree with 100 leaves and generated nucleotide sequences of length 1000 by evolving them on the tree. Figure 2 illustrates the performance of the **l**-method, i.e., MAP over lengths, relative to the **r **× **t**-method and the combined method, i.e., MAP over rates and times without and with DP, respectively. We note that finding **r **and **t **that optimize the prior probabilities requires more than 200,000 iterations for the **r **× **t**-method to converge although the data probability has reached optimum after only 50,000 iterations approximately. The speedup achieved with the combined method, as compared to the **r **× **t**-method, is considerable. We note also that the combined algorithm obtains a solution with higher log-likelihood than does the otherwise fast **l**-method. When the performances of these methods are compared on a larger set of trees, the conclusions stated above become even clearer. We generated 100 trees with 10 leaves and 100 trees with 100 leaves, and generated nucleotide sequences of length 1000 on each tree. For each of our three methods, we further made two separate parameter estimations on each tree. The aim was to compare the two results and from that evaluate the respective methods' reproducibility. The results in Table 1 show the methods' performances with respect to optimality, speed, and reproducibility of likelihood, rates, and times. On average the combined method delivers the result with best likelihood, the **r **× **t**-method is second, while it is again clear that the **r **× **t**-method is by far the slowest. We note in particular that the **l**-methods' rate and time variance is almost negligible; but this is simply a consequence of the fact that the **l**-method consistently finds very similar **l**-optima, and that the optimal DP-partitioning of these **l**-optima results in very similar **r **and **t**. To summarize, the combined method outperforms the others with respect to quickly finding optimal values for branches' rates and times.

**Table 1:** Parameter inference simulations. Results from pairwise MAP runs on 100 generated tree topologies using the combined method, the r × t-method, and the l-method.

Tree size	**Method**^1^	MAP value	**MAP diff**^2^		**Rates diff**^3^		**Times diff**^4^		**Comp time**^5^
		average	average	worst	average	worst	average	worst	relative
10 leaves	combined	-8046.54	0.48	3.39	0.58	1.46	0.26	0.72	1
	**r **× **t**	-8046.72	0.32	13.03	0.36	4.61	0.17	1.73	2
	**l**	-8049.34	0.012	0.20	0.00068	0.0084	0.0015	0.05	1
100 leaves	combined	-65249.12	1.61	8.60	4.72	10.64	1.24	3.39	2
	**r **× **t**	-65257.40	4.66	23.10	14.08	21.66	5.11	9.19	10
	**l**	-65258.41	0.16	1.54	0.034	0.071	0.060	0.41	1

^1^The combined method and the **r **× **t**-method infer optimal substitution rates and divergence times. The **l**-method infer optimal branch lengths which are subsequently partitioned into rates and times. The latter problem is much easier and the inferred rates and times less optimal.

^2^Difference in log-likelihood between two MAP runs on the same tree.

^3^Difference in substitution rate norm between two MAP runs on the same tree.

^4^Difference in divergence time norm between two MAP runs on the same tree.

^5^Approximate computation time relative to the **l**-method.

### Evaluation of parameter recovery

To get an indication of what accuracy we can expect for solutions obtained with the hill-climbing approach, we compared the inferred rates and times parameters with the corresponding values used when simulating the sequence data. We generated 10 trees with 10 leaves and 10 trees with 100 leaves, then generated nucleotide sequences of length 1000 on each tree. We recorded the true rates and times for each edge, and we ran the combined method and the **r **× **t**-method on each tree while recording the rates and times thereby inferred. We repeated the procedure using different values of *v*, the rate variance hyperparameter, when generating the sequence data. The rate mean hyperparameter was kept constant at 0.5.

**Figure 3:**
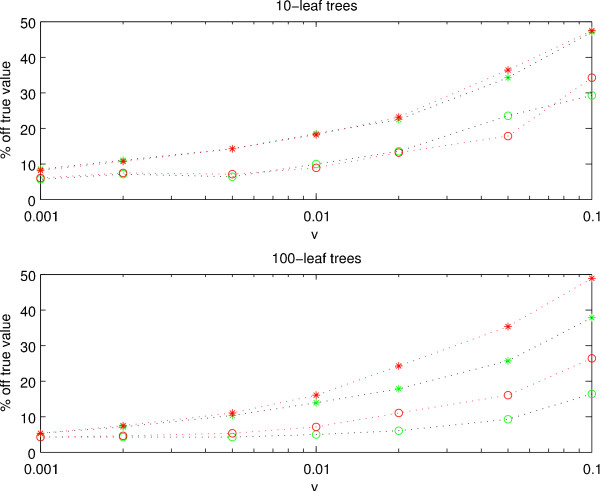
**Degree of parameter recovery**. Rates and times inferred using the combined method (green curves), and the **r **× **t**-method (red curves) are compared with the corresponding true parameters that were used to generate the sequence data. Results for 10-leaf (upper figure), and 100-leaf trees (lower figure) are shown. We plot the deviation of the inferred parameter from the true value against different rate variances *v*. Rates and times are shown as stars and circles, respectively.

From Figure 3 can be seen that for 10-leaf trees and *m *= 0.5, *v *= 0.001, the inferred rates and times are on average around 5% off the true value. For these *m *and *v *the quotient between the highest and the lowest true rate in the tree is approximately 1.25, showing that the data is fairly clock-like. For *m *= 0.5, *v *= 0.1, on the other hand, the rate parameters are as much as 45% off and the time parameters around 30% off. Here, though, the highest true rate is more than 5 times greater than the lowest. The same pattern is seen also for large 100-leaf trees, with the accuracy being very high for fairly clock-like data and declining as the data becomes less clock-like. Interestingly, we note that for the 100-leaf trees, the combined method is much more accurate than the **r **× **t**-method with respect to both rates and times. With the combined method, the times, in particular, and the rates, are very well estimated even for *v *as large as 0.05.

### Hill-climbing vs. MCMC – a parameter inference performance comparison on a rbcL dataset

**Figure 4:**
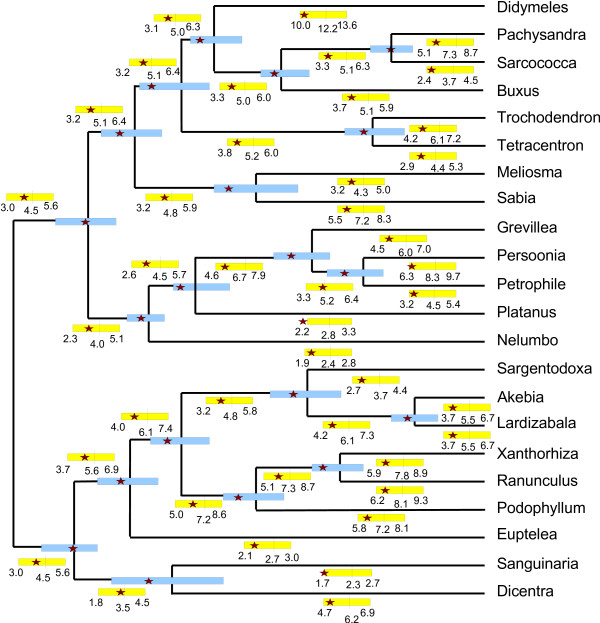
**An rbcL hill-climbing vs. MCMC comparison**. The speciation times inferred by using MCMC are shown by the tree nodes (means) and the blue bars (25% and 75% quantiles), respectively. The positions of the tree nodes, and the 25% and 75% bars, are relative to the position of the root, which has time value 1.0. Substitution rates are explicitly written out scaled by 100 on branches and are shown by yellow bars (mean, 25% and 75% quantiles). In both cases, corresponding MAP estimates are indicated by red stars.

We also made a parameter inference comparison on a dataset consisting of *rbcL *genes from *eudicots*, a group of flowering plants. We used the tree presented in [28]. We compared the rates and times inferred using the combined method, i.e., hill-climbing MAP with the DP factorization speedup, with the rates and times posterior distributions obtained from an MCMC analysis using the **r **× **t**-method (i.e. without DP). The MCMC-chain was run 100,000,000 iterations, and was sampled at regular intervals (Sennblad et al.: Parental guidance vs. mutual independence – evaluation of bayesian models of substitution rate evolution, submitted). To evaluate the convergence of the MCMC analysis, the Gelman and Rubin [29] convergence tests, as implemented in the *R *package *Coda *[30], was used. The MCMC-analysis required a computation time of several days, while the MAP-optimum is reached after some 10.000 steps, requiring less than a minute on the same type of computer, the difference mainly being due to MCMC requiring more iterations to accurately describe the posterior distribution while MAP results in point estimates. Figure 4 depicts the rates and times posterior distributions inferred using MCMC, together with the corresponding values from the MAP-analysis. We note that for the 62 rate and time variables that we attempt to infer, only one has a MAP estimate outside of the borders marked out by the 25% and 75% quantiles from the MCMC analysis, and that one is only slightly outside. This is good performance, since it seems clear from the figure that the MCMC analysis gives rather tight parameter inference, i.e., the quantile intervals are small.

### Phylogeny inference simulations

**Figure 5:**
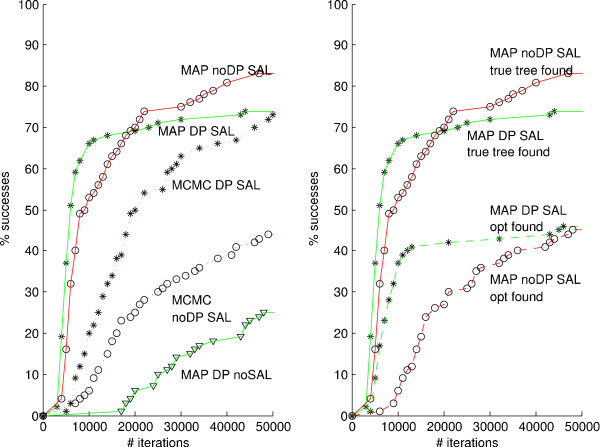
**Phylogeny inference simulations**. Phylogeny inference simulations performed on 30-leaf trees are shown. An inference is considered successful at the first visit to the true tree, i.e., the tree generating the sequence data, or to a state with log-likelihood value at least as good as the true tree. In the left figure, we plot percent successes as a function of number of iterations for 100 runs on one tree with, respectively, MAP using the combined method, i.e., with DP (solid green line with stars indicating that at least one run has reached the success zone during the last 1000 steps), MAP using the **r **× **t**-method, i.e., without DP (solid red line with circles) and MCMC with (dotted green line with stars) and without DP (dotted red line with circles). We also plot results obtained with MAP and the combined method but without the SAL-method (solid green line with triangles). In the right figure, the comparison is between the same MAP runs with (again solid green line with stars) and without DP (again red solid line with circles) when a success is recorded as before, and the same methods (dashed green and red lines with stars and circles respectively) where a success is recorded when *optimum *for the true tree is reached.

Compared to the parameter inference problem discussed in the previous three sections, the phylogeny inference problem is significantly more difficult. Given a set of sequences, the objective is to perform simultaneous inference of tree topology, rates, and times. In Figure 5 and Table 2, we present a simulation study comparing hill-climbing and MCMC, with the result, as expected, that hill-climbing converges quickly but is less reliable than MCMC. In both the hill-climbing and the MCMC case, a run is considered successful when it first visits a state with log-likelihood at least as good as the optimal log-likelihood for the tree generating the data. For MCMC this is despite the fact that if the posterior distribution is non-uniform it cannot have been reached. That is, we underestimate the time required.

**Table 2:** Phylogeny inference simulations. 100 times MAP and MCMC phylogeny inference on 10- and 30-leaf trees, using the combined, the *r *× *t-method*, the standard and the simulated annealing-like algorithms respectively.

		Tree size	**# Iterations**^1^		**Time**^2^		**Success**^3^	
			SAL	Std	SAL	Std	SAL	Std
Combined		10	2000	1500	50 s	15 s	98	87
	MAP	30	6000	29000	11 m	20 m	74	25
		10	4200	1700	90 s	20 s	99	100
	MCMC	30	18000	32000	20 m	25 m	80	60
**r **× **t**		10	1900	3300	45 s	25 s	99	98
	MAP	30	8000	>50000	14 m	>30 m	83	0
		10	5000	4600	100 s	30 s	96	100
	MCMC	30	22000	>50000	20 m	>30 m	54	0

^1^Median number of iterations for successful runs (i.e. runs finding a tree with likelihood at least as good as the true tree).

^2^Median computation time needed for successful runs.

^3^Percentage of runs being successful.

When doing phylogeny inference the local optima problem really becomes an issue. What often happens both in the hill-climbing MAP and in the MCMC case, is that the algorithm gets stuck on one particular sub-optimal tree, and by optimizing rate and time parameters for that tree we make a move to another tree unlikely. To circumvent this problem, we use the SAL-method described in Methods; that is, we do tree swaps often early in the chain, while focusing more on fine-tuned parameter changes later. For small trees the effect of the SAL-method is minor. Out of the 100 MAP estimations we carried out for the 10-leaf trees with the SAL-method and the standard method (i.e. perturbing each parameter equally often), we finished successfully in 98 and 87 cases, respectively (see Table 2). When using MCMC on 10-leaf trees, we finished successfully in 99 cases with the SAL-method and in 100 cases with the standard method. For 10-leaf trees, both the standard method and the SAL-method require a similar number of iterations to converge successfully, and we conclude that the difference in computation time between the two methods for these small trees is minor.

For inference on larger trees (results from simulations using 30-leaf trees are shown), it is clear that favoring of early tree moves, as in the SAL-method, is necessary. Out of 100 simulations that were performed with the standard method, after 50,000 iterations only 25 MAP runs have reached a likelihood similar to that of the true tree (i.e. the tree generating the data). This should be compared with the SAL-method which finishes successfully in 74% of the cases. Also for MCMC, where we expect optimal values to be eventually achieved in all cases, we note the same pattern with the SAL-method having much shorter time to success. We finally conclude that the effect of our DP-algorithm is less pronounced when it comes to phylogeny inference than is the case for parameter inference. A comparison between the combined method (i.e. with the DP augmentation) and the **r **× **t**-method is shown in the right part of Figure 5. We record the percentage of runs finding first the true tree and second the optimal log-likelihood for that tree. The combined method finds the true tree in 74% and the **r **× **t**-method in 83% of the cases. The combined method is slightly faster needing on average 6000 iterations while the **r **× **t**-method needs 8000 iterations. Now, to find the optimum the combined method needs 8000 iterations, i.e., another 2000 iterations after the true tree is found. This should be compared to the **r **× **t**-method which needs 8000 iterations to find the optimum after the true tree is found, in total 16000 iterations on average.

### Hill-climbing vs. MCMC – a phylogeny inference performance comparison on a mtDNA dataset

**Figure 6:**
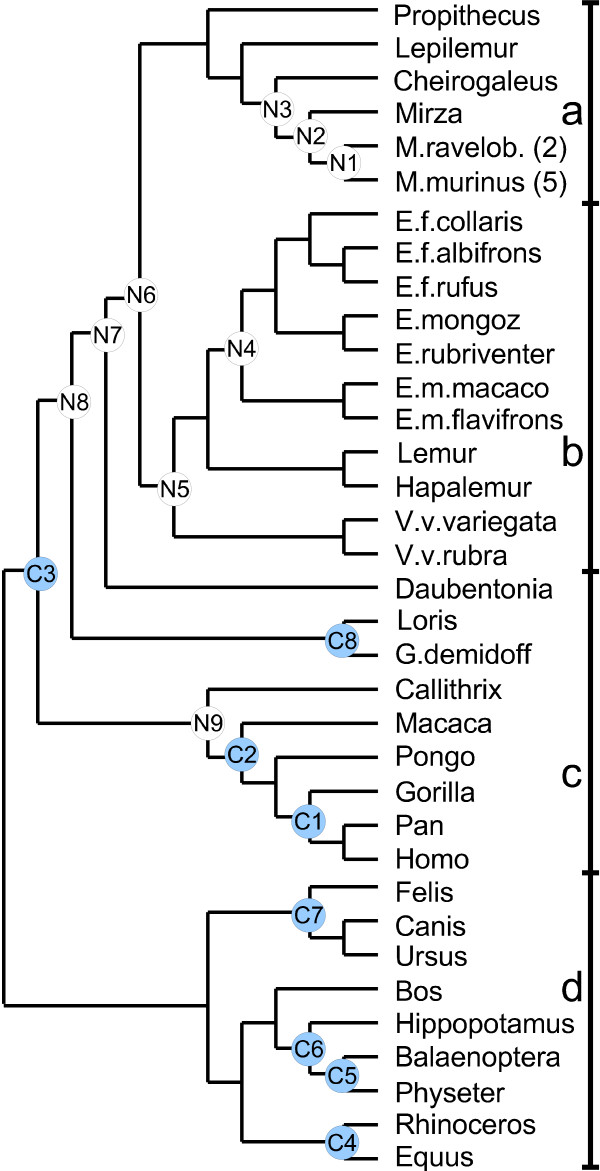
**Malagasy lemur phylogenetic tree**. Phylogenetic tree used for divergence time inference in [23]. The upper part of the tree (categories **a **and **b**) contains the Malagasy lemurs, which are the main objects of analysis in the article, while the lower part (sections **c **and **d**), is for calibration purposes. We report our inference on branching times shown with blue circles C1–C8, these are calibration points in [23], and on branching times shown with white circles N1–N9, these are inferred also in [23].

To test the phylogeny inference method on biological data, we used a mitochondrial DNA dataset originally presented in [23]. It consists of the complete *cytochrome oxidase II *and *cytochrome b *genes, altogether around 1800 nucleotides, in 40 species. The authors utilize this and other datasets together with calibration times obtained from fossil records to infer divergence times among the lemurs of Madagascar. The model they used is that of Thorne *et al. *[14] which, again, is based on auto-correlated rates. Our use of this data was twofold. Firstly, we tested whether, and if so how often, we could find a tree with similar or higher likelihood than the one used in [23] (see Figure 6). Secondly, we used their tree in order to obtain divergence times for comparison.

We partitioned the sequence set into four categories **a**, **b**, **c**, **d **as shown in Figure 6. We ran our tree inference on successively larger proportions of the tree, i.e., subtrees **a**, **a **+ **b**, **a **+ **b **+ **c**, and the full **a **+ **b **+ **c **+ **d **tree. On each subtree, we recorded optimal log-likelihood values for the tree as given in the figure. We then ran 100 separate inferences starting from a random tree to see whether the obtained log-likelihood values differed much mutually and whether we could reach the optimal value for the given tree. Note that, in Table 3, the success percentage varies over trees in an seemingly unexpected manner. We thus record a higher percentage of successes for the dataset including 31 sequences in trees **a **+ **b **+ **c **than for the smaller subset in trees **a **+ **b **(22 sequences) and **a **(11 sequences). A plausible reason for this is that the tree used to compare with (i.e. the one shown in Figure 6) is not necessarily optimal and that the relative effect of this is larger for the smaller subtrees. The distribution of results inferred by our method seems however to be the expected one with wider spread for bigger trees.

**Table 3:** Phylogeny inference performed on a mtDNA dataset. 100 times MAP phylogeny inference on step by step larger proportions of the tree shown in Figure 5.

	**Known**^1^	**Inferred**^2^			**Success**^3^
Tree	tree	best	median	worst	%
**a**	-8843.8	-8816.8	-8829.0	-8953.7	72
**a **+ **b**	-13381.9	-13345.0	-13369.6	-13472.5	65
**a **+ **b **+ **c**	-22054.2	-21977.7	-22025.0	-22339.3	73
**a **+ **b **+ **c **+ **d**	-30327.7	-30235.7	-30315.5	-30658.7	58

^1^Reference log-likelihood value obtained with the tree topology in Figure 5.

^2^Log-likelihood value obtained starting from a random tree topology.

^3^Percentage of runs finding a tree with likelihood at least as good as for the tree topology in Figure 5.

### Hill-climbing vs. MCMC – a dating performance comparison on a mtDNA dataset

We finally made a comparison between divergence times resulting from our method and those reported in [23]. The results in Table 4 imply that our inference differs considerably from the calibration times used by Yoder *et al.*. All times inferred by us on the *Laurasiatheria *clade (C4–C7) are wildly underestimated as compared to the reported calibrations, although the relative edge lengths on the clade seem to agree. On the other hand, we overestimate the time point for the divergence between *Pan/Homo *and *Gorilla *(C1). Our results agree with what has been reported by Arnason *et al. *on several occasions, [31] is a recent example, from studies on primate mtDNA data. It is possible that the discrepancy between our results and those presented by Yoder *et al. *stems from the fact that we do not include the additional information that calibration times give.

**Table 4:** Dating analysis performed on a mtDNA dataset. Comparing our method's divergence times estimations with the calibration points reported by Yoder *et al. *(C1–C8 upper section), and inference points (N1–N9 lower section) estimates.

		C1	C2	**C3**^1^	C4	C5	C6	C7	C8
Yoder *et al. *– mean – 95% interval		10.9^2 ^(8.9,12.0)	34.0 (32.1,37.5)	85.9 (78.4,89.9)	53.0 (50.1,57.5)	34.8 (33.1,38.7)	56.0 (51.4,59.8)	58.1 (48.9,64.6)	40.0 (38.1,41.9)
Our inference		20.7^3^	38.5	-	31.6	18.8	30.6	40.5	37.5

	**N1**	**N2**	**N3**	**N4**	**N5**	**N6**	**N7**	**N8**	**N9**

Y – mean Y – 95%	12.0^4 ^(7.8,17.9)	24.2 (16.8,33.4)	31.8 (23.4,41.6)	8.4 (5.3,13.4)	35.9 (27.0,46.3)	46.7 (36.9,57.5)	67.1 (56.8,77.2)	72.9 (64.0,82.0)	61.8 (51.0,73.8)
Our inference	14.8^5^	24.7	32.6	15.8	39.5	50.4	64.2	71.1	60.2

^1^Reference node for our estimation.

^2^MYA calibration for **C1 **reported by Yoder *et al.*

^3^MYA estimation for **C1 **by our method.

^4^MYA estimation for **N1 **reported by Yoder *et al.*

^5^MYA estimation for **N1 **by our method.

More to the point, out of the nine inferred time points (N1–N9), we are inside the reported 95% credibility interval on all occasions but one (the *Eulemur *divergence time point N4 being the exception), and in most cases we are close to the means reported by Yoder *et al.*. This is obtained from an inference using a mere 40 seconds computation time.

## Discussion

We have presented new methods for dating analysis and phylogenetic tree estimation using nucleotide sequence data. We use a model with birth-death priors on tree branching and iid substitution rates among lineages, originally developed in a Markov chain Monte Carlo (MCMC) framework (Sennblad et al.: Parental guidance vs. mutual independence – evaluation of bayesian models of substitution rate evolution, submitted), enabling simultaneous inference of substitution rates and divergence times. We show that use of this model in a maximum *a posteriori *(MAP) framework strongly improves the opportunities to perform biologically relevant analyses on a large scale.

In addition to this, we have developed a DP algorithm intended to meet the computationally challenging problem of optimally partitioning branch lengths into rates and times. This contribution, which works as nicely in an MCMC as in a MAP framework, limits the computation time of our MAP-algorithm to nearly that of standard ML phylogenetic inference, i.e., where one does not bother about separating branch lengths into rates and times but only infers the lengths.

The possibility to simultaneously estimate rates and times in an efficient way is of great interest in comparative genomics, as well as in both systematic and evolutionary biology. Moreover, the current usage of uniform priors on branch lengths has recently been shown to be problematic [17]. Modeling branch substitution rates and divergence times separately allows us to use a birth-death prior on the times and still evade a molecular clock.

Until now, maximum *a posteriori *has not been used in this context. It might be that the computational difficulties linked with inference of an optimum in the huge rates and times space, have hampered the development of MAP-algorithms for this problem. Instead, the present trend in the phylogenetic inference field of leaving the strict molecular clock has mainly included MCMC-based methods. This is a class of methods benefiting both from a natural way of including biologically motivated prior beliefs and a natural way of expressing uncertainties in the solutions; but on the negative side must be counted the relative slowness of these methods. Compared to MCMC methods, our hill-climbing algorithm has significant computational advantages. First, by accepting only moves increasing the total log-likelihood, our method generally finds a local optimum quicker than does the MCMC variant. Second, since we only seek one particular value, i.e., the locally optimal point, we naturally know when to stop the search and we can do without the sampling needed for MCMC-methods to ensure acceptable mixing.

A significant drawback of our method is that it only delivers point estimates of the inferred variables. The standard procedure when inferring tree topologies, nonparametric bootstrapping, cannot easily be implemented in the context presented here. It is probably possible to retrieve uncertainty estimates for a specified time point of interest. One could fix one interval below and one above the MAP estimate and calculate the summed probability of positioning the node in question in that interval. It is, however, not clear how such an uncertainty estimate would compare to the MCMC equivalent.

We have further shown that, for the hill-climbing algorithm, the all-important local optima problem can be addressed. For parameter inference on a fixed tree, where this issue is not as problematic, we believe that a comparison between results from multiple runs starting from different, randomly selected, positions, will most often be sufficient. The problem gets more noticeable when inferring (large) phylogenies. We have tested our method on simulated datasets using trees with 30 leaves and biological datasets using trees of similar size. We have noted that there is a risk of optimizing parameters for one specific tree topology so much that escaping from that tree is made difficult. This was not unexpected, however, we found that it can often be avoided by using the SAL-method, a scheme where tree topologies are swapped relatively more often in the beginning of the inference chain.

The gain of using our methodology, instead of standard ML, for topology inference is twofold. First, if the sequences at hand are of short or moderate length, the influence of the rates and times prior will be considerable, favoring a well-chosen prior distribution. Second, when fossil data include known time values, our methodology is the natural choice, since these data can easily be included with our methodology, but not so in an ML inference. If neither of these two apply, the natural procedure is to use ML for topology inference, followed by our methodology for inference of rates and times parameters.

We have noted that when inferring trees with very short edges it would be advantageous for us to locally use a dense grid for the edge times. Similarly when doing phylogeny inference it might be of interest to use the DP-algorithm more often right after a tree swap in order to get the factorization of lengths into rates and times correct before investigating the new tree. These are possible directions for further investigations. In the presented analyses on simulated data, we have worked with predefined hyperparameter values for the rate and time priors. Such values are generally not known for biological data, for which we instead estimate these values using maximum likelihood. It is straight-forward to extend our method to include estimation of the hyperparameters. We have noted that our method's performance is data-sensitive in the sense that highly non-clocklike data is not handled well and that varying rate hyperparameters might have an influence on results. We suspect from previous results [15,21,32] that the influence of the time prior will show that the effect of its hyperparameters on the MAP-estimates will be low. Further studies on this aspect would, however, be interesting. Another interesting aspect is the influence of the priors for long sequences. It is clear [32,33] (Sennblad et al.: Parental guidance vs. mutual independence – evaluation of bayesian models of substitution rate evolution, submitted) that the width of the rates and times posterior intervals will decrease for longer sequences but that this is only true up to a point. Even for infinitely long sequences there will be uncertainties in these estimations.

## Conclusion

Our contribution leaves the field open for fast and accurate dating analysis of nucleotide sequence data. Compared to MCMC-methods, our methodology reduces inference time for large phylogenies by orders of magnitude. Our novel DP-algorithm is an integral part of the methodology and simulation-based comparisons between our combined method and the **r **× **t**-method, i.e., hill-climbing with and without the DP-algorithm, show that the DP-algorithm is most rewarding when difficult problems are attacked. For inference on very large trees it delivers both speed and accuracy. Also, the DP-algorithm is superior when the sequence data at hand are non-clocklike.

The method is easily adapted to take divergence time information into account, e.g. from fossil data [34,35], by restricting a speciation to a specific interval or using a prior distribution on the interval. By including priors on branching times we have introduced irreversibility into our model which at least in principle could be used for tree rooting. Since standard ML phylogenetic models are reversible with respect to time, one has to resort to information outside of the model, normally an outgroup sequence, for positioning of the root. An investigation of whether we can do better in this regard is an interesting direction for future research.

## Availability and requirements

Project name: PRIME – Probabilistic Integrated Models of Evolution Project home page: http://prime.sbc.su.se/map_dp Operating systems: Linux, MacOSx Programming language: C++ Licence: Source code not released yet. Binaries are available on the project home page. Restrictions for non-academic use: None

## Authors' contributions

ÖÅ and JL planned the study and wrote the paper. ÖÅ and BS wrote the C++ code for simulating and analysing the data. BS and ÖÅ retrieved the biological data. All authors have read and approved the final version of the manuscript.
